# Fluorescent Oxygen-Doped g-C_3_N_4_ Quantum Dots for Selective Detection Fe^3+^ Ions in Cell Imaging

**DOI:** 10.3390/nano12111826

**Published:** 2022-05-26

**Authors:** Jiahui Zhang, Yan Jing, Peng Zhang, Benhua Xu

**Affiliations:** 1 Qinghai Provincial Key Laboratory of New Light Alloys, Qinghai Provincial Engineering Research Center of High-Performance Light Metal Alloys and Forming, Qinghai University, Xining 810016, China; z276475670@126.com; 2 Chemical Engineering College, Qinghai University, Xining 810016, China; jingyan@163.com (Y.J.); xubenhua@qhu.edu.cn (B.X.)

**Keywords:** oxygen-doped g-C_3_N_4_ quantum dots, fluorescence, metal ion detection, bioimaging

## Abstract

Herein, oxygen-doped g-C_3_N_4_ quantum dots (OCNQDs) were fabricated through sintering and ultrasonic-assisted liquid-phase exfoliation methods. The obtained OCNQDs with uniform size show high crystalline quality, and the average diameter is 6.7 ± 0.5 nm. Furthermore, the OCNQDs display excellent fluorescence properties, good water solubility, and excellent photo stability. The OCNQDs as fluorescence probe show high sensitivity and selectivity to Fe^3+^ ions. Furthermore, the fluorescent OCNQDs are applied for live cell imaging and Fe^3+^ ions detecting in living cells with low cytotoxicity, good biocompatibility, and high permeability. Overall, the fluorescent OCNQDs fabricated in this work can be promising candidates for a range of chemical sensors and bioimaging applications.

## 1. Introduction

Fe^3+^ is one of the most fundamental trace metal cations in the human body, which exhibits many critical physiological functions such as electron transfer, metabolic reactions, and neuroregulation [1,2]. However, abnormal levels of Fe^3+^ in the body can destroy the physiological functions of organisms, which can cause several of diseases involving anemia, insomnia, and some iron metabolism disorder diseases [3,4]. Therefore, developing economical and accurate methods to detect Fe^3+^ in various liquid samples, as well as in living cells is necessary. To date, due to the disadvantages such as complexity, time-consuming, valuableness, and tedious preparation, the traditional analytical approaches are limited to detect metal ions economically and effectively in aqueous solution and living cells [5,6,7,8]. Therefore, the fluorescent probe has caught widespread attention as a simple, low-cost, and nontoxic sensing platform [9,10,11,12]. Among them, metal-free quantum dot-based fluorescent nanoprobes are appropriate for biosensing and bioimaging due to the merits of low toxicity and no secondary contamination during applications.

g-C_3_N_4_ quantum dots (gCNQDs) are the typical representative of metal-free quantum dots and have demonstrated advantages of fascinating optical properties, good biosafety, good water solubility, and easy functionalization, which enable gCNQDs unprecedented opportunities as fluorescent probe for metal ions detecting and bioimaging [13,14,15]. To date, the gCNQDs obtained by sonicating 2D g-C_3_N_4_ (gCN) nanosheets and 1D gCN nanowires have been developed to detect Cu^2+^ and Fe^3+^ [16,17]. In order to further expand the fluorescence performance of gCN, the amount of functionalization strategies involving doping and surface functionalization have been exploited. For example, gCN can be modified with organic ligands, micromolecules, organic dyes [18,19,20], and carboxyl [21]. gCN can be doped with nonmetal and metal elements [22]. Hence, gCNQDs could be functionalized via doping and surface functionalization. For instance, P-doped gCN nanosheets prepared by ultrasonic stripping was used as efficient fluorescent probe for Fe^3+^ detection [23]. Carboxyl-rich gCN nanoparticles were synthesized through one-pot thermal polymerization method and were used as a fluorescent probe for Hg^2+^ and Fe^3+^ detecting [21]. However, the application of doped gCNQDs as fluorescence probes for chemical sensors and bioimaging has been rarely reported. Therefore, it is necessary to prepare doped gCNQDs and investigate their fluorescence properties for the applications of biosensing and bioimaging.

Herein, we report a simple method to synthesize OCNQDs. The obtained OCNQDs with an average diameter of 6.7 ± 0.5 nm show high crystalline quality and excellent fluorescence properties, good water solubility, and excellent photo stability. The fluorescent OCNQDs can be sensitive and selective fluorescence probe for Fe^3+^ detecting. Moreover, the fluorescent OCNQDs with low cytotoxicity, good biocompatibility, and high permeability are applied for live cell imaging and Fe^3+^ ions detecting in living cells.

## 2. Experimental Section

### 2.1. Materials

Melamine, ethanol (EtOH), and *N*,*N*-dimethylformamide (DMF) were obtained from Aladdin Chemistry Co., Ltd. (Shanghai, China). H_2_O_2_ (30 wt%) was purchased from Sinopharm Chemical Reagent Co., Ltd. (Shanghai, China).

### 2.2. Apparatus

The scanning electron microscope (SEM, JSM-6610LV, JEOL Ltd., Tokyo, Japan) was used to investigate the morphologies of samples. The transmission electron microscope (TEM, JEM-2100F, JEOL Ltd., Tokyo, Japan) was used to investigate the morphologies and structures of samples. The crystallographic information of the samples was characterized using an X-ray diffractometer (XRD, D8 FOCUS, Bruker, Karlsruhe, Germany). The vibrational spectra of samples were probed with Fourier transform infrared spectroscopy (FT-IR BXII, Perkin-Elmer, Waltham, MA, USA). The element identification was measured using an X-ray photoelectron spectrometer (XPS, ESCALAB Xi+, Thermo Fisher Scientific, Waltham, MA, USA). The UV–vis spectra and fluorescence performances were probed using a spectrophotometer (Cary Series UV–Vis–NIR, Agilent Technologies, Beijing, China) and spectrophotometer (Cary Eclipse fluorescence, Agilent Technologies, Beijing, China). All pH measurements were made using a pH-10C digital pH meter.

### 2.3. Synthesis of Bulk gCN

The bulk gCN was synthesized via calcining of melamine. Specifically, 5 g of melamine was calcined in the muffle furnace at 600 °C for 2 h under air atmosphere with a heating rate of 5 °C/min. Then, yellow bulk gCN was synthesized after natural cooling to 20 °C.

### 2.4. Synthesis of Bulk O-Gcn

First, 7.2 g of melamine was dispersed in mixture solution of 5 mL H_2_O_2_ and 35 mL deionized water by ultrasonic treatment for 30 min. Then, the obtained suspension was transferred into a 100 mL Teflon-lined stainless autoclave. After maintaining at 140 °C for 12 h, the autoclave was naturally cooled to 20 °C. After that, the white precipitate was obtained through repeated high-speed centrifugal cleaning with deionized water and absolute ethanol. The above-obtained melamine precursor dried in a vacuum drying oven was sintered at 600 °C for 2 h in air. Then, yellow bulk O-gCN was synthesized after natural cooling to 20 °C.

### 2.5. Synthesis of OCNQDs

0.5 g of O-gCN powders was dispersed in DMF by ultrasonicating for about 30 min, and the obtained suspension was sonicated by the ultrasonic cell disruption system at 200 W for 2 h. Afterward, the mixture of O-gCN and OCNQDs were obtained by concentrating using rotary evaporator at 80 °C. The mixture was dispersed in 1000 mL deionized water and sonicated for 1 h. After using fractional centrifugation, amounts of OCNQDs were obtained. Then, via centrifuging at 10,000× *g* for 8 min, the yellow suspension containing abundant OCNQDs was obtained. Eventually, OCNQDs powders were obtained by vacuum freeze dryer at −60 °C.

### 2.6. Photoluminescence Measurement

The concentration of the prepared OCNQDs was adjusted to 10 μg/mL in deionized water. The stock solutions of the metals (Fe^3+^, Fe^2+^, Na^+^, Li^+^, Ba^2+^, K^+^, Ag^+^, Cd^2+^, Zn^2+^, Al^3+^, Pb^2+^, Mn^2+^, Hg^2+^, Cr^3+^, Cu^2+^, Co^2+^, Ni^2+^, Mg^2+^, and Ca^2+^) were prepared using perchlorate of metal ions to be 1 M in ultrapure water. Herein, 2 mL of OCNQDs solution was mixed with 3 μL of solution containing each metal ion. After incubation at room temperature, the fluorescence performances of the OCNQDs were investigated under excitation at 320 nm.

### 2.7. Cell Culture and Viability/Cytotoxicity Assay

A549 cells (American Type Culture Collection, Manassas, VA, USA) were cultured using in Dulbecco’s modified Eagle medium containing 10% fetal bovine serum in 5% CO_2_ humidified incubator at 37 °C. Subsequently, the cells were washed with phosphate-bufered saline for three times. MTT assay was performed to investigate the cytotoxicity of the A549 cells after the incubation with the OCNQDs. The cells were treated with different concentrations of OCNQDs (0 μg/mL, 3.125 μg/mL, 6.25 μg/mL, 12.5 μg/mL, 25 μg/mL, 50 μg/mL, 100 μg/mL, and 200 μg/mL) for 24 h.

## 3. Results and Discussion

**Figure 1:**
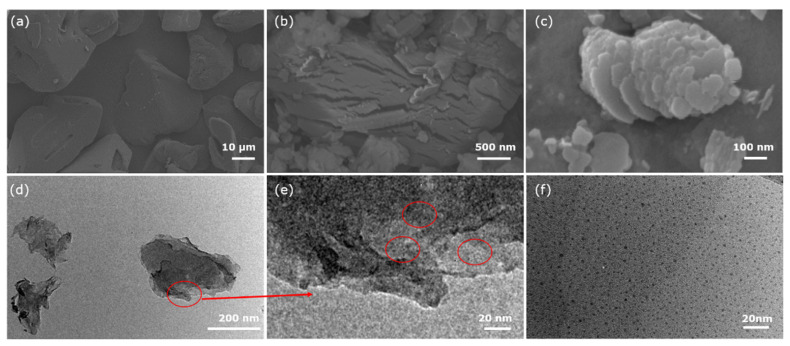
The SEM images of (**a**) melamine, (**b**) melamine precursor, (**c**) bulk O-gCN; the TEM images of (**d**) O-gCN nanosheets, (**e**) the edge of O-gCN nanosheets at higher magnification, (**f**) OCNQDs.

The morphologies of the samples were characterized by SEM and TEM. As shown in Figure 1a, original melamine powders are comprised of irregular particles with smooth surface and the particle size is in the range from 10 μm to 100 μm. After the hydrothermal treatment of melamine in H_2_O_2_ solution, the surface of melamine precursor is chapped and the particles size is reduced to several micrometers, as depicted in Figure 1b. Furthermore, the hydrothermal treatment contributes to doping oxygen atoms in melamine precursor. Then, the bulk O-gCN was obtained by calcining the obtained oxygen-doped melamine precursor in air. As shown in Figure 1c, the bulk O-gCN exhibits the morphology of stack of plates, which helps to synthesize the O-gCN quantum dots through reducing the size of bulk O-gCN less than 10 nm by ultrasonic-assisted liquid-phase exfoliation via the ultrasonic cell disruption system. Figure 1d shows the TEM image of O-gCN nanosheets obtained by ultrasonic treatment and amount of OCNQDs are adherent to the surface of O-gCN nanosheets (Figure 1e). Finally, OCNQDs were obtained by using fractional centrifugation, as presented in Figure 1f.

**Figure 2:**
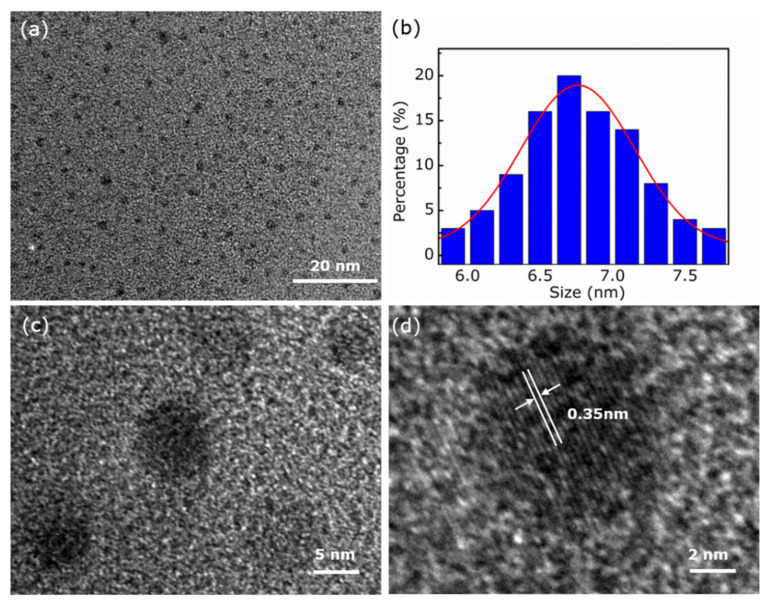
(**a**) TEM image, (**b**) particle size distribution analysis, (**c**,**d**) HRTEM images of OCNQDs.

The morphologies and size distribute of OCNQDs dispersed in water were performed using the high-resolution TEM (HRTEM). As displayed in Figure 2a, OCNQDs possess good monodisperse and uniform size. The particle size is range from 5 to 8 nm with an average diameter of 6.7 nm, as shown in histogram of size distribution (Figure 2b). The HRTEM image exhibits the OCNQDs with paralleled and ordered lattice fringes. The lattice d-spacing of the OCNQDs is 3.5 Å, which is assigned to (002) face. To sum up, the prepared OCNQDs with uniform size are monodispersed.

**Figure 3:**
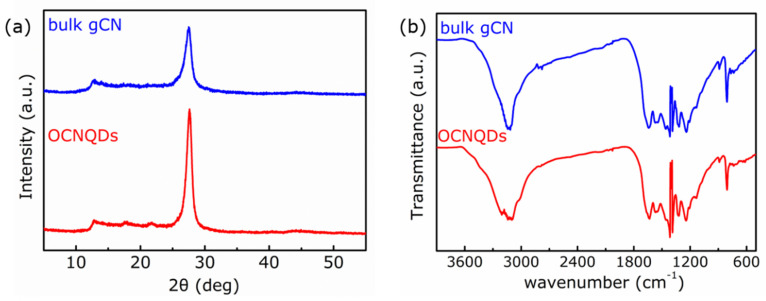
(**a**) The XRD diagrams; (**b**) the FT-IR spectra of bulk gCN and OCNQDs.

The crystal structures of bulk gCN and OCNQDs were studied by XRD. Figure 3a presents that the two characteristic peaks at 13.2° and 27.4° belong to (100) and (002) planes of bulk gCN [24,25]. The XRD patterns of bulk gCN and OCNQDs are similar, which means the crystal structure of bulk gCN is maintained during the size reducing from tens micrometers to several nanometers. The (002) peak in XRD pattern of the OCNQDs shifts from 27.4° to 27.7° with the interplanar stacking distance decrease [26]. When the C or N atoms in tri-s-triazine motifs are replaced by O, the O doping in gCN can improves H_2_O_2_ the electronegativity of the graphitic framework, which strengthens the interaction between the gCN layers and leads to shorten the interplanar distance [27]. FTIR spectra of bulk gCN and OCNQDs were presented in Figure 3b. The similar FTIR spectra for bulk gCN and OCNQDs display the characteristic peaks at 809 cm^−1^ and 1200–1700 cm^−1^ are from tri-s-triazine units and nitrogen-containing groups [28]. In the range 3000–3500 cm^−1^, a broadband belonging to the (N–H) or (O–H) vibrations mode, which may originate from the surface uncondensed amine groups or the absorbed water species, is observed [29]. Compared with bulk gCN, the intensity of these peaks of OCNQDs decreases evidently, resulting from triazine ring breaking. The above results suggest that OCNQDs was successfully synthesized through replacing C or N atom in gCN by O atoms [30,31].

**Figure 4:**
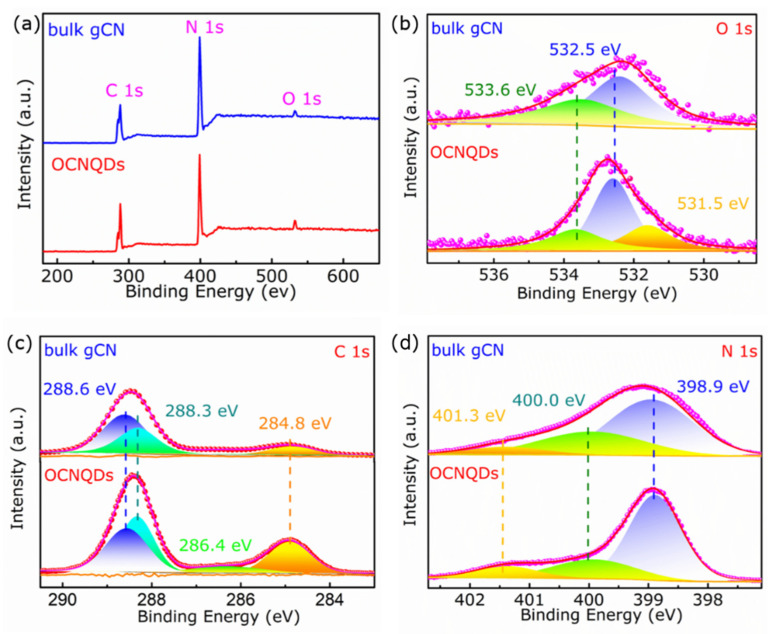
(**a**) The survey XPS spectra; (**b**–**d**) narrow scan spectra of O 1s, C 1s, and N 1s of OCNQDs and bulk gCN.

The chemical composition of the OCNQDs and bulk gCN was characterized by the XPS. As the survey spectra exhibited in Figure 4a, the OCNQDs and bulk gCN are composed of C, N, and O elements. As shown in Figure 4b, two peaks located at 533.6 eV and 532.5 eV are observed in the O 1s high-resolution spectrum, which are ascribed to the absorbed oxygen specie and water from air. Furthermore, three peaks located at 531.5, 532.5, and 533.6 eV in O 1s high-resolution spectrum are assigned to the C–O bonding, oxygen species, and water. The appearance of C–O bonding confirms the successful synthesis of OCNQDs [32,33]. As shown in Figure 4c, three peaks located at 284.8, 288.3, and 288.6 eV in C 1s spectrum of bulk gCN are assigned to C–C/C=C, N–C=N [34] and O–C=O bonding [35]. Furthermore, in C 1s high-resolution spectrum of OCNQDs, the new peak located at 286.4 eV assigning to C–O bonding is observed. The generation of C–O bonds in OCNQDs demonstrates that O atoms are doped into gCN by substituting N atoms [32]. In the high-resolution N 1 s spectrum of bulk gCN (Figure 4d), the peaks at 398.9, 400.0, and 401.3 eV can be assigned to the C–N–C, C–N=C, and quaternary nitrogen [36,37]. Meanwhile, the elemental analyses performed by XPS prove that the O atoms in OCNQDs have observably increased (Table 1). Furthermore, the C/N ratio of OCNQDs is higher than that of bulk gCN, which also confirms that O atoms are doped into gCN and OCNQDs are successfully obtained.

**Figure 5:**
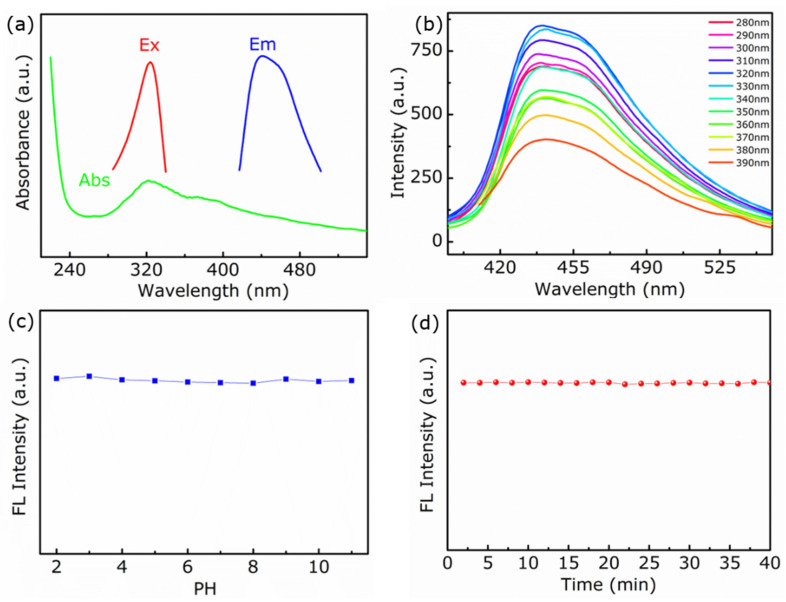
(**a**) UV–visible absorption and emission spectrum of OCNQDs. The excitation and emission wavelengths are 440 nm and 320 nm. (**b**) Fluorescence spectra at different excitation wavelengths ranging from 280 nm to 390 nm. The effect of (**c**) pH and (**d**) irradiation on the fluorescence intensity of OCNQDs.

The optical properties of the OCNQDs were researched extensively. As exhibited in the UV–vis spectra of OCNQDs (Figure 5a), a characteristic absorption peak located at 320 nm was observed, which is contributed by π–π* electronic transitions for gCN containing s-triazine rings [16]. The maximum fluorescence emission peak of OCNQDs was available at 440 nm with excitation wavelength at 320 nm. Figure 5b shows the typical photoluminescent (PL) spectra of OCNQDs. With increasing excitation wavelength from 280 to 390 nm, the PL spectra are independent of the excitation wavelengths, which are dominated by surface states mainly [38]. Moreover, it is crucial to investigate the photo-stability of OCNQDs for biosensing and bioimaging applications. Figure 5c depicts the effect of pH on the fluorescence performances of OCNQDs. With the pH values increasing from 2 to 12, no significant interference of pH with OCNQDs is found, indicating the pH-independent behavior. Moreover, when OCNQDs undergo continuous irradiation for 40 min, the fluorescence intensity of OCNQDs does not have apparent variation, showing high resistance of OCNQDs to photobleaching. Meanwhile, the absolute quantum yield of OCNQDs is 5.49%. The above results demonstrate that the obtained OCNQDs display fluorescence properties, good water solubility, and photostability, contributing them to apply in biosensing and bioimaging.

**Figure 6:**
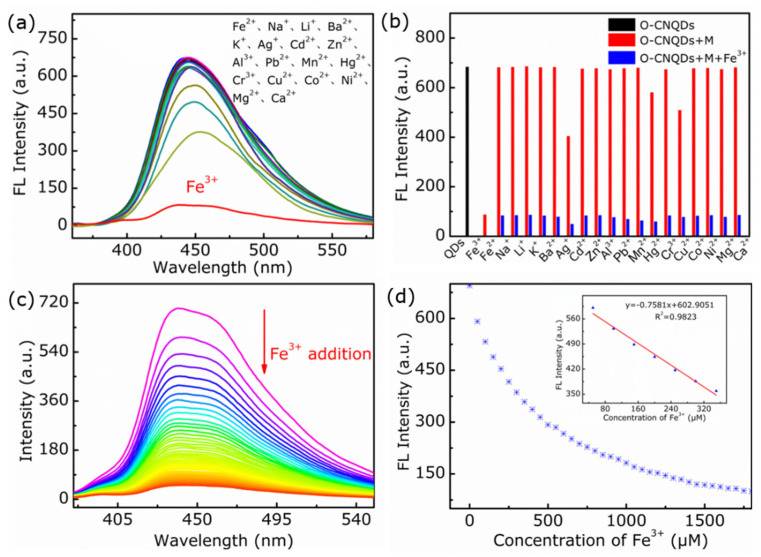
(**a**,**b**) Fluorescence intensity of OCNQDs in aqueous solution containing various metal ions (1500 μM for Fe^3+^, Fe^2+^, Na^+^, Li^+^, Ba^2+^, K^+^, Ag^+^, Cd^2+^, Zn^2+^, Al^3+^, Pb^2+^, Mn^2+^, Hg^2+^, Cr^3+^, Cu^2+^, Co^2+^, Ni^2+^, Mg^2+^, and Ca^2+^). (**c**) Fluorescence spectra of OCNQDs upon addition of various concentrations of Fe^3+^. (**d**) Relationship between fluorescence intensity of OCNQDs and concentration of Fe^3+^. Inset: the linear relationship of fluorescence intensity of OCNQDs versus the concentration of Fe^3+^ over the range from 0.1 to 340 μM.

Generally, the fluorescence intensity of QDs can be changed by metal ions. The change of fluorescence signal of OCNQDs generated by various metal ions could be used to make OCNQDs as a fluorescent probe for effective metal ion sensor. As shown in Figure 6a, various metal ions (Fe^3+^, Fe^2+^,Li^+^, Ba^2+^, Na^+^, K^+^, Ag^+^, Zn^2+^, Al^3+^, Cd^2+^, Pb^2+^, Mn^2+^, Hg^2+^, Cr^3+^, Cu^2+^, Co^2+^, Ni^2+^, Mg^2+^, and Ca^2+^) are added independently in OCNQDs aqueous suspensions and the fluorescence intensity of OCNQDs is significantly decreased by Fe^3+^, implying that the OCNQDs could be selected as a fluorescent probe for the sensitive detection of Fe^3+^. Then, the selectivity of OCNQDs for Fe^3+^ was investigated. As shown in Figure 6b, when metal ions besides Fe^3+^ were added into OCNQDs aqueous solution, fluorescence intensity of OCNQDs is basically stable. Then, the fluorescence can be quenched when the Fe^3+^ and other metal ions are added into OCNQDs aqueous solution simultaneously, indicating the fine selectivity for Fe^3+^. To evaluate the sensing ability of probe for Fe^3+^, the PL-dependent emission spectra on Fe^3+^ is performed. As shown in Figure 6c, with increasing the concentration from 0 μM to 1500 μM, the fluorescence intensity of OCNQDs at 440 nm is gradually decreased. The prepared OCNQDs exhibit linear detection of Fe^3+^ in the range of 1–400 μM with a limit of detection (LOD) of 2.47 μM, which is estimated from the linear equation: detection limit = 3σ/k [39]. The LOD value is lower than permissible concentration of Fe^3+^ (0.3 ppm) in water recommended by WHO [40].

**Figure 7:**
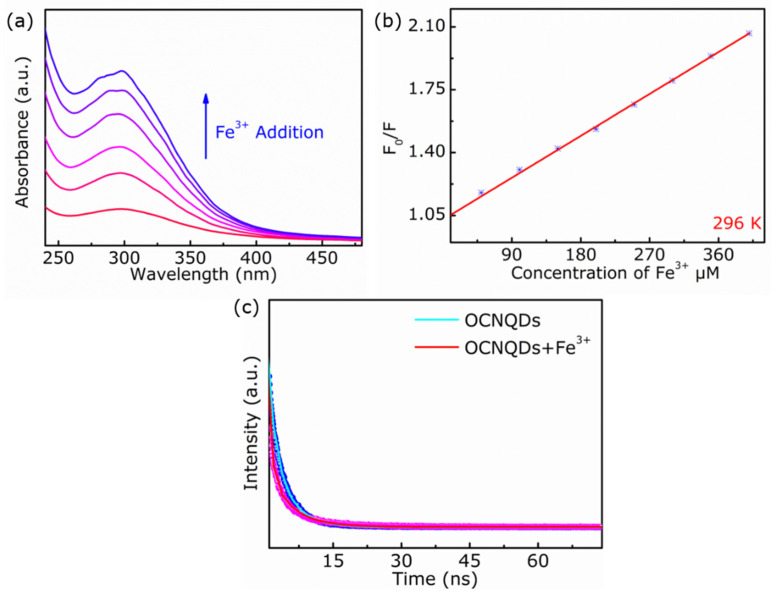
(**a**) The UV–vis spectra of OCNQDs in the absence and presence of Fe^3+^ (0–3000 μM). (**b**) Stern–Volmer plots describes the dependency of the fluorescence intensities on the Fe^3+^ concentration over the range of 0–400 μM. (**c**) Fluorescence lifetime of OCNQDs in the presence or absence of Fe^3+^.

Figure 7a shows that the absorption peak at 300 nm in UV–vis spectra of OCNQDs increase dramatically with the addition of Fe^3+^, implying that the possible quenching mechanism is static quenching [41]. The possible quenching mechanism is studied by the Stern–Volmer equation: F_0_/F = K_SV_[Q] + 1 [42], where F_0_ and F are the fluorescence emission intensities in the presence and in the absence of Fe^3+^, respectively. K_SV_ is the Stern–Volmer constant, and [Q] is the concentration of Fe^3+^. The obtained data is fitted in the Stern–Volmer equation as: F_0_/F = 2.59 × 10^3^ [Fe^3+^] + 1.0266 (R^2^ = 0.9979), K_SV_ is 2.59 × 10^3^ L/mol (Figure 7b). Furthermore, the quenching rate constant (k_q_) is calculated by the equation: k_q_ = 1 + K_SV_/τ_0_, where τ_0_ is the fluorescence lifetime of OCNQDs without of Fe^3+^ [43]. The values of k_q_ (4.45 × 10^11^ L/mol/s) is larger than the maximum collision quenching constant (2.0 × 10^10^ L/mol/s) for the quenching fluorescence molecules, suggesting a more probable static quenching. Moreover, the fluorescence lifetimes of the OCNQDs with or without Fe^3+^ are used to get deep understanding of the quenching mechanism. As shown in Figure 7c, the average fluorescence lifetime of OCNQDs varies from 5.82 to 5.71 and shows no obvious change, which further confirmed the static quenching. The above results further demonstrate that the fluorescence quenching provoked by Fe^3+^ is static quenching.

**Figure 8:**
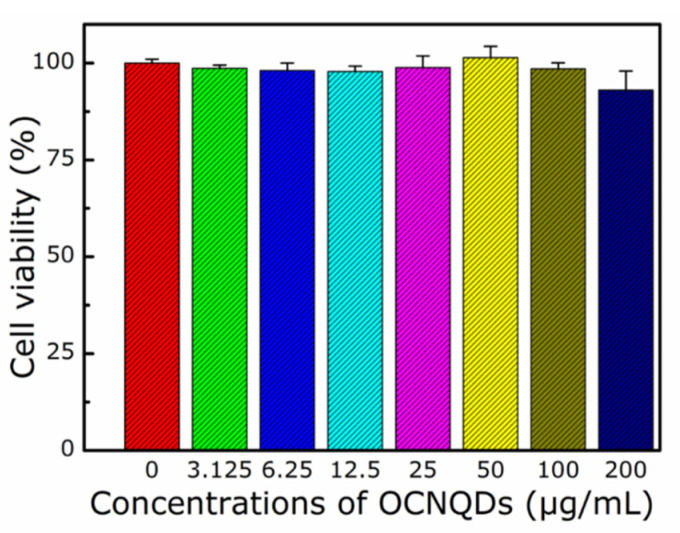
Cell viability of A549 cells after incubation with OCNQDs at varying concentrations of 24 h using an MTT assay.

To demonstrate the cytotoxicity of the obtained OCNQDs, the MTT assay was carried out. The A549 cells were incubated with the OCNQDs at varying concentrations of 24 h. As shown in Figure 8, the cells were treated with different concentrations of OCNQDs (0 μg/mL, 3.125 μg/mL, 6.25 μg/mL, 12.5 μg/mL, 25 μg/mL, 50 μg/mL, 100 μg/mL, and 200 μg/mL) and cell viability was decreased slightly with the increasing concentrations. Moreover, the survival rates of the cells approached 93% when the concentration of OCNQDs reached to 200 µg/mL. These results confirm the cytotoxicity and biocompatibility of the OCNQDs toward the cells, providing an advantage for cell imaging and other potential biological applications.

**Figure 9:**
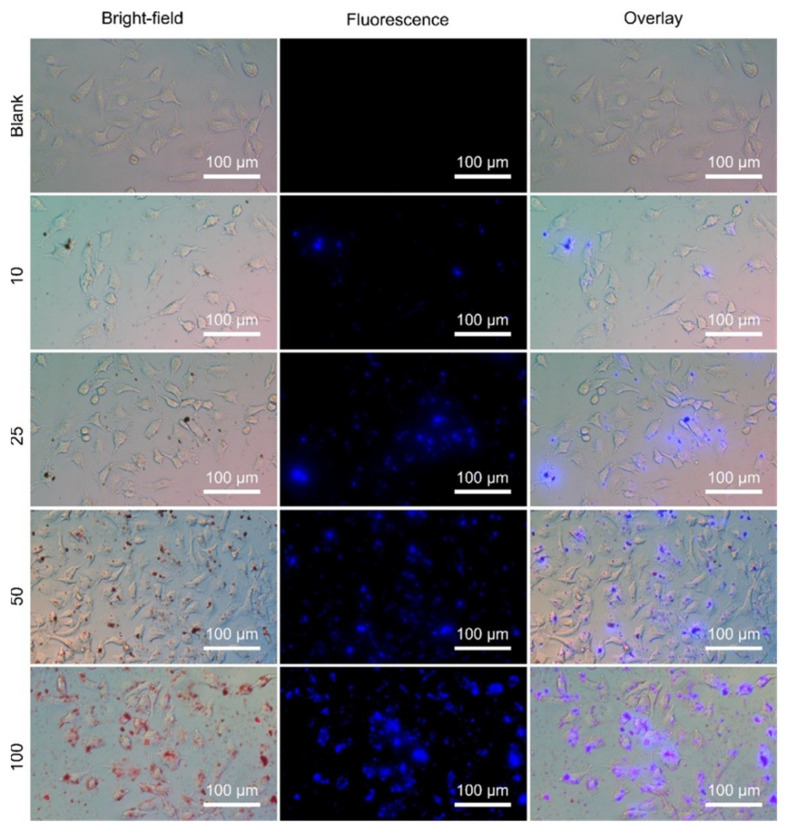
Confocal images of A549 cells incubated with OCNQDs (0 μg/mL, 10 μg/mL, 25 μg/mL, 50 μg/mL, and 100 μg/mL) for 2 h at 37 °C.

In Figure 9, fluorescence microscopy images of A549 cells treated with OCNQDs for 2 h at 37 °C are presented. The blue fluorescence becomes brighter with the increase of the OCNQDs concentrations from 10 μg/mL to 100 μg/mL and the morphologies of the A549 cells basically remain.

**Figure 10:**
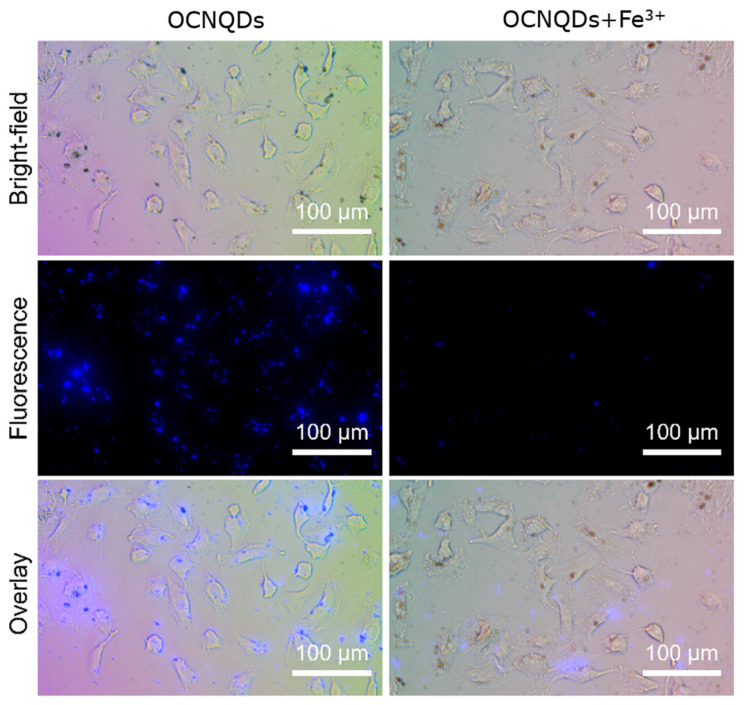
Bright-field, fluorescence, and overlay images of A549 cells incubated with OCNQDs (25 µg/mL) for 4 h at 37°.

In order to use the fluorescence sensor for the detection of Fe^3+^ in living cells, cellular imaging was evaluated by confocal laser scanning microscopy. As shown in the bright-field images (Figure 10), the morphology of A549 cells have not been affected after the addition of OCNQDs (25 µg/mL) for 4 h at 37 °C, which indicated that the OCNQDs had excellent cell permeability. As expected, the A549 cells treated with OCNQDs showed bright blue fluorescence under 320 nm laser excitation exhibits of a fluorescence microscope. However, the bright blue fluorescence is completely quenched when the cells are treated with OCNQDs (25 µg/mL) and further incubated with Fe^3+^ (1000 µM) for another 30 min. These observations clearly indicate that the fluorescence probe OCNQDs can be used in biological detection of Fe^3+^.

## 4. Conclusions

A combination of sintering and ultrasonic-assisted liquid-phase exfoliation method was exploited to synthesize the OCNQDs. The obtained OCNQDs display high crystalline quality and fluorescence properties, good water solubility, and photostability. The fluorescent OCNQDs can be sensitive and selective fluorescence probe for Fe^3+^, which are utilized for the fluorescence imaging of Fe^3+^ in living cells with low cytotoxicity, good biocompatibility, and high permeability.

## Figures and Tables

**Table 1:** Elemental analysis (C, N, O atomic %) of the OCNQDs and bulk gCN performed by XPS.

Materials	C (Atomic %)	N (Atomic %)	O (Atomic %)	C/N (Atomic Ratio)
OCNQDs	42.41	51.67	5.92	0.82
bulk gCN	41.10	56.39	2.51	0.73

## Data Availability

Not applicable.
